# Uncertainty Characterisation of Mobile Robot Localisation Techniques using Optical Surveying Grade Instruments

**DOI:** 10.3390/s18072274

**Published:** 2018-07-13

**Authors:** Benjamin J. McLoughlin, Harry A. G. Pointon, John P. McLoughlin, Andy Shaw, Frederic A. Bezombes

**Affiliations:** Engineering and Technology Research Institute, Liverpool John Moores University, 3 Byrom St, Liverpool L3 3AF, UK; h.a.pointon@2016.ljmu.ac.uk (H.A.G.P.); j.mcloughlin@ljmu.ac.uk (J.P.M.); a.shaw@ljmu.ac.uk (A.S.)

**Keywords:** robotic total station, localisation, ultra wide-band, extended Kalman filter, RTS

## Abstract

Recent developments in localisation systems for autonomous robotic technology have been a driving factor in the deployment of robots in a wide variety of environments. Estimating sensor measurement noise is an essential factor when producing uncertainty models for state-of-the-art robotic positioning systems. In this paper, a surveying grade optical instrument in the form of a Trimble S7 Robotic Total Station is utilised to dynamically characterise the error of positioning sensors of a ground based unmanned robot. The error characteristics are used as inputs into the construction of a Localisation Extended Kalman Filter which fuses Pozyx Ultra-wideband range measurements with odometry to obtain an optimal position estimation, all whilst using the path generated from the remote tracking feature of the Robotic Total Station as a ground truth metric. Experiments show that the proposed method yields an improved positional estimation compared to the Pozyx systems’ native firmware algorithm as well as producing a smoother trajectory.

## 1. Introduction

Localisation is a fundamental aspect in the area of mobile robotics. The ability for a robot to relate itself to an operating environment, a global frame of reference and other robotic systems is a key challenge in the development and deployment of autonomous systems.

In order to achieve autonomy, a mobile unmanned robot must be equipped with a localisation or positioning system that consistently and precisely determines robot pose, i.e., position and heading, as it navigates throughout an environment. Localisation methods and techniques are classified into two categories, relative and absolute. Relative localisation methods are generally conducted within the body frame of the platform, through the integration of techniques such as vision based odometry systems without image georeferencing [1,2], dead reckoning via inertial measurement units (IMU) [3,4] and wheel odometry (in the case of ground based systems) to determine the speed of the robotic platform [5,6]. Absolute techniques refer to the localisation of co-ordinate reference frames that are external to the robot, for example the use of independent landmarks [7,8], known correspondence reference points [9] or a Global Navigation Satellite System (GNSS) [10]. However, common implementations include the collaboration of relative and absolute positioning systems [11,12]. This combination is usually integrated within state estimation algorithms such as Particle Filters (PF) and both linear and nonlinear Kalman Filters, where such multi-sensor fusion techniques are designed whilst considering motion models and robot idiosyncrasies [13].

Considering outdoor environments, one notable example and a frequently implemented localisation technique is the use of GNSS and, when combined with an inertial navigation system (INS), GNSSs are often implemented as a standard technique for many commercial and off-the-shelf unmanned systems. For example, GNSS on unmanned aerial vehicles (UAVs) are often operated in conjunction with high resolution cameras for image georeferencing and photogrammetry techniques [14]. The implementation of the differential variant of GNSS for 3D light detection and ranging (LiDAR) systems for point cloud registration and georeferenced map generation has also been investigated [15]. However, the performance of GNSS in areas of mountainous terrain and urban locations with abounding tall structures as well as indoor environments have been a reason for others to explore alternative localisation techniques [16,17]. For this work, an alternative method for localisation in the form of Ultra-wideband (UWB) is investigated.

### 1.1. Ultra-Wideband Localisation Systems

UWB is a wireless radio technique that transmits and receives narrow pulses at a nanosecond rate. Unlike conventional radio frequency identification (RFID) based systems that operate on single bands, UWB transmits over a broad spectrum of radio frequencies [18]. The ability to transmit over this extensive bandwidth decreases the power spectral density, therefore enabling UWB based systems to avoid interfaces with other RF signals [19,20]. Range measurements produced from UWB are achieved from the accurate resolution of the Time of Arrival (TOA), commonly known as Time of Flight (TOF), of a pulsed waveform that is traveling between a target node (tag) and set of reference nodes (anchors) [21]. Furthermore, as a result of the large operating bandwidth, the UWB system obtains a high time resolution; therefore, positioning techniques can provide accurate range estimations [21]. Due to the aforementioned capabilities, UWB positioning systems have previously been applied to human tracking [22], sporting scenarios [23] and engineering applications such as the direct geo-referencing of images from an aerial platform [24]. Static characterisation of range uncertainty behaviour between two static UWB nodes has been previously investigated [25]. In order to calculate the range uncertainty for the work carried out in [25], static range readings were taken at various distances between two nodes and compared to a true distance for error calculations. Although this method allows static based range error characterisation, the method does not provide a dynamic error reading of the UWB system under the intended operating conditions of the deployed system. However, to obtain a more accurate judgment of the expected range error, the system would need to be monitored over various types of movement in continuous rather than discrete samples.

Deploying robotic systems with a means to make judgments and interact with the surrounding environment is entirely dependent upon an imperative element of robotics, which is uncertainty [9]. Uncertainty can arise from areas such as unpredictable environments, actuation factors, inaccurate system modeling and noise due to onboard sensors. However, for the case of this study, the range uncertainty of an network of sensor nodes from a UWB localisation system is characterised using a surveying grade optical instrument in the form of a Robotic Total Station (RTS).

### 1.2. Robotic Total Stations

A Trimble S7 RTS (Sunnyvale, CA, USA) was used as the principal method for uncertainty estimation and final system appraisal in the experiments. In normal operation, the RTS system is used in the laying out of building works, inspection of existing and ongoing building works and surveying of land. The RTS takes measurement readings through the use of an optical and remote distance sensing unit with an expected accuracy of 2 mm + 2 ppm in standard prism mode and 4 mm + 2 ppm in tracking prism mode depending on the prism in use [26]. In the case of Electronic Distance Measurement (EDM) devices, the accuracy of a measurement is dependent on a number of factors. However, an estimate of the error may be achieved through the use of the stated accuracy specifications over an example distance of 1 km such [27]:

(1) $$\pm (2\;\mathrm{mm}+\left(2\;\mathrm{ppm}\times \left(1\times 10{}^{6}\;\mathrm{mm}\right)\right)=\pm 4\;\mathrm{mm}.$$

Therefore, the expected measurement error during this study may be taken as the upper bounding error found at the maximum operating distance of 20 m. This error may be taken as ±2.04 mm. The bearing of the target is taken through the use of an absolute encoder whose accuracy may be defined within DIN 18723, the specification for theodolite accuracy from the German Institute for Standards, at 1” (arc seconds) or 2.77 × 10${}^{-6}$ degrees standard deviation [26]. The range that the RTS is capable of tracking over depends on the prism or target in use, but, for the prism used in this experiment, distances of up to 500 m could be measured.

The main functionality of the UWB system is to dynamically localise the platform that is mounted with the mobile tag. Therefore, utilising an RTS with active target tracking features provides a foundation to characterise the range uncertainty of the robot in its intended operating conditions as opposed to a static based test. Using the RTS as a ground truth comparison metric to dynamically characterise the uncertainty of a UWB sensor network for state estimation system design is an unexplored area of research. There has also been little investigation into the use of such techniques in the field of mobile robot positioning using UWB systems. Work has been conducted that studies the implementation of an RTS system as a ground truth for the assessment of the validity of other localisation systems [28]. Another study to note conducted by [29] focuses on using UWB for position estimation of a large scale laser scanning unit for 3D Building Information Modeling (BIM). Additionally, a further analysis has been conducted into the viability of using UWB systems to track operatives in construction sites, and this investigation utilised an RTS for the ground truth [30]. The aforementioned investigations demonstrated the viability in applications; however, the uncertainty and lack of state estimation made the implementation of such a system inviable for applications requiring high positional accuracy. Other work carried out using an RTS to actively track unmanned systems consists of autonomous and remote positioning of a Micro Aerial Vehicle (MAV) using real-time input from the RTS [31]. An RTS has also been deployed as a substitute for GNSS as a measurement input source into an Extended Kalman Filter (EKF) for indoor environments [32].

Therefore, this paper seeks to utilise an RTS as a technique to determine the uncertainty of a UWB positioning system, as well as the uncertainty of a set of quadrature wheel encoders used on a mobile robotic platform. These uncertainty metrics are used as an input into an EKF for recursive state estimation to improve the overall position and orientation estimation, otherwise known as the pose of the robot.

## 2. State Estimation Formulation

### 2.1. Problem Formulation

A discrete EKF was used as the sensor fusion object to combine the initial position estimation from a control input with external range measurement updates acquired from the UWB anchors. The EKF calculates a linear approximation for a set of nonlinear functions based on the first-order Taylor expansion [9]. This is achieved through a two-stage iteration process where the future state of the system is predicted based on the current state and a state transition model. This is then corrected using a measurement from an external source. The nonlinear state transition and measurement models for an EKF are described in Equations (2) and (3) where $\hat{X_{k}}$ and $\hat{Z_{k}}$ represent the state and measurement vector estimations at time *k*, controlled input into the system is represented as $u_{k}$ and notations $w_{k-1}$ and $v_{k-1}$ are the system and measurement noise:

(2) $$\hat{X_{k}}=f\left(X_{k-1},u_{k};w_{k-1}\right),$$

(3) $$\hat{Z_{k}}=h\left(X_{k-1},v_{k-1}\right).$$

For the system implemented in this study, the range measurements acquired from the UWB anchors meant that the system was assessed as a range-based localisation problem. This problem when concerning mobile robots includes the continuous estimation of the robot’s state in terms of its planar Cartesian coordinates and heading $X={\left[x\;y\;\phi \right]}^{T}$ [33], in relation to the reference anchors in the environment that are assumed to have static positions mapped in $M_{\mathrm{Bi}}=\left[x_{\mathrm{Bi}}\;y_{\mathrm{Bi}}\right],i\in \left[1,\ldots ,N_{B}\right]$ and provide a set of range measurements $Z_{\mathrm{Bi}}=\left[r_{\mathrm{Bi}},i\in \left[1,\ldots ,N_{\mathrm{Bi}}\right]\right]$ at time *k*, where $N_{B}$ is the number of UWB anchors present. The EKF was used to integrate the initial position estimate with the external range measurements acquired from the UWB beacons. The EKF model used for localisation is based on that presented by Thrun et al. [9]. In the case of this study, sensor measurements with an initial state prediction were based on systematic mathematical modeling fused with with a control odometry input, where quadrature encoders are used to determine the angular displacement of the robots wheels at time *k* as well as a time synchronised gyroscope for heading calculation. The designs of the formulation for the state transition and measurement model are presented below.

#### 2.1.1. State Transition Model

An input odometry model proposed by [13] and also adopted by [25] was chosen to formulate the state transition of the robot:

(4) $${\hat{X}}_{k}^{-}=f\left({\hat{X}}_{k-1},u_{k}\right),$$

(5) $$f\left({\hat{X}}_{k-1},u_{k}\right)={\hat{X}}_{k-1}+\left[\begin{array}{c} \delta D_{k}\cos\left(\phi_{k}+\frac{{\delta \phi }_{k}}{2}\right)\\ \delta D_{k}\sin\left(\phi_{k}+\frac{{\delta \phi }_{k}}{2}\right)\\ \phi_{k}+\delta \phi_{k} \end{array}\right],$$

where $\delta D_{k}$ is the linear displacement at time k and is calculated using the radii of the wheels $r_{L}$, $r_{R}$ and the angular displacement $\theta_{L_{k}}$ and $\theta_{R_{k}}$ acquired from the pulses of the encoders:

(6) $$\delta D_{k}=\frac{r_{L}\delta \theta_{L_{k}}+r_{R}\delta \theta_{R_{k}}}{2}.$$

Therefore, with the state vector defined as $X={\left[x\;y\;\phi \right]}^{T}$, the input vector is represented as $u_{k}={\left[\theta_{L}\;\theta_{R}\;\delta_{\phi }\right]}^{T}$, enabling the system and input Jacobians $G_{x}$ and $G_{u}$ to be pre-formulated.

(7) $$G_{x_{k}}=\frac{\partial f}{\partial x}\left({\hat{X}}_{k}^{-},u{}_{k}\right),$$

(8) $$G_{x_{k}}=\left[\begin{array}{ccc} 1 & 0 & -\frac{\delta D_{k}\;\sin\left(\phi_{k}+\frac{\delta \phi_{k}}{2}\right)}{2}\\ 0 & 1 & \frac{\delta D_{k}\;\cos\left(\phi_{k}+\frac{\delta \phi_{k}}{2}\right)}{2}\\ 0 & 0 & 1 \end{array}\right],$$

(9) $$G_{u_{k}}=\frac{\partial f}{\partial u}\left({\hat{X}}_{k}^{-},u{}_{k}\right),$$

(10) $$G_{u_{k}}=\left[\begin{array}{ccc} \frac{r_{L}\;\cos\left(\phi_{k}+\frac{\delta \phi_{k}}{2}\right)}{2} & \frac{r_{R}\;\cos\left(\phi_{k}+\frac{\delta \phi_{k}}{2}\right)}{2} & -\frac{D_{k}\;\sin\left(\phi_{k}+\frac{\delta \phi_{k}}{2}\right)}{2}\\ \frac{r_{L}\;\sin\left(\phi_{k}+\frac{\delta \phi_{k}}{2}\right)}{2} & \frac{r_{R}\;\sin\left(\phi_{k}+\frac{\delta \phi_{k}}{2}\right)}{2} & \frac{D_{k}\;\cos\left(\phi_{k}+\frac{\delta \phi_{k}}{2}\right)}{2}\\ 0 & 0 & 1 \end{array}\right].$$

#### 2.1.2. Measurement Model

The observed sensor measurements collected from UWB devices are range measurements representing the Euclidean distances between the static reference anchors $A_{B_{i}}$ and the estimated position of the robot. At each measurement update, six anchors each provided a range measurement $Z_{\mathrm{Bi}}=\left[z_{1},z_{2}\ldots z_{6}\right]$, which is represented within the measurement function *h* below. Uncertainty within the measurements is represented by noise variance *R*. An overview of the EKF algorithm is shown in Algorithm 1:
**Algorithm 1** Range based EKF Localisation   **Prediction:**1:${\hat{X}}_{k}^{-}=f\left({\hat{X}}_{k-1},u_{k}\right)$2:$G_{x_{k}}=\frac{\partial f}{\partial x}\left({\hat{X}}_{k}^{-},u{}_{k}\right)$3:$G_{u_{k}}=\frac{\partial f}{\partial u}\left({\hat{X}}_{k}^{-},u{}_{k}\right)$4:${\hat{P}}_{k}^{-}=G_{x_{k}}{\hat{P}}_{k-1}G_{x_{k}}^{T}+G_{u_{k}}QG_{u_{k}}^{T}$   **Correction:**5:${\hat{Z}}_{Bi}=h\left({\hat{X}}_{k}^{-},A_{B_{i}}\right)$6:$H_{z_{k}}=\frac{\partial h}{\partial x}\left({\hat{X}}_{k}^{-}\right)$7:$K_{k}={\hat{P}}_{k}^{-}H_{z_{k}}{\left(H_{z_{k}}{\hat{P}}_{k}^{-}H_{z_{k}}^{T}+R\right)}^{-1}$8:$y=Z_{Bi}-{\hat{Z}}_{Bi}$9:${\hat{X}}_{k}={\hat{X}}_{k}^{-}+K_{k}y$10:${\hat{P}}_{k}=\left(I-K_{k}H_{z_{k}}\right){\hat{P}}_{k}^{-}$11:**if** ! measurement_is_available **then**12:  do **Prediction**13:**else**14:  do **Correction**15:**end if**

(11) $${\hat{Z}}_{Bi}=h\left({\hat{X}}_{k}^{-},A_{B_{i}}\right),$$

(12) $$h\left({\hat{X}}_{k}^{-},A_{B}\right)=\left[\begin{array}{c} \sqrt{{\left(x_{k}-x_{b1}\right)}^{2}+{\left(y_{k}-y_{b1}\right)}^{2}}\\ \sqrt{{\left(x_{k}-x_{b2}\right)}^{2}+{\left(y_{k}-y_{b2}\right)}^{2}}\\ \sqrt{{\left(x_{k}-x_{b3}\right)}^{2}+{\left(y_{k}-y_{b3}\right)}^{2}}\\ \sqrt{{\left(x_{k}-x_{b4}\right)}^{2}+{\left(y_{k}-y_{b4}\right)}^{2}}\\ \sqrt{{\left(x_{k}-x_{b5}\right)}^{2}+{\left(y_{k}-y_{b5}\right)}^{2}}\\ \sqrt{{\left(x_{k}-x_{b6}\right)}^{2}+{\left(y_{k}-y_{b6}\right)}^{2}} \end{array}\right],$$

and the Jacobian of the measurement model is obtained as:

(13) $$H_{z_{k}}=\frac{\partial h}{\partial x}\left({\hat{X}}_{k}^{-}\right),$$

(14) $$H_{z_{k}}=\left[\begin{array}{ccc} \frac{x_{k}-x_{b1}}{\sqrt{{\left(x_{k}-x_{b1}\right)}^{2}+{\left(y_{k}-{\mathrm{yb}}_{1}\right)}^{2}}} & \frac{y_{k}-y_{b1}}{\sqrt{{\left(x_{k}-x_{b1}\right)}^{2}+{\left(y_{k}-y_{b1}\right)}^{2}}} & 0\\ \frac{x_{k}-x_{b2}}{\sqrt{{\left(x_{k}-x_{b2}\right)}^{2}+{\left(y_{k}-y_{b2}\right)}^{2}}} & \frac{y_{k}-y_{b2}}{\sqrt{{\left(x_{k}-x_{b2}\right)}^{2}+{\left(y_{k}-y_{b2}\right)}^{2}}} & 0\\ \frac{x_{k}-x_{b3}}{\sqrt{{\left(x_{k}-x_{b3}\right)}^{2}+{\left(y_{k}-y_{b3}\right)}^{2}}} & \frac{y_{k}-y_{b3}}{\sqrt{{\left(x_{k}-x_{b3}\right)}^{2}+{\left(y_{k}-y_{b3}\right)}^{2}}} & 0\\ \frac{x_{k}-x_{b4}}{\sqrt{{\left(x_{k}-x_{b4}\right)}^{2}+{\left(y_{k}-y_{b4}\right)}^{2}}} & \frac{y_{k}-y_{b4}}{\sqrt{{\left(x_{k}-x_{b4}\right)}^{2}+{\left(y_{k}-y_{b4}\right)}^{2}}} & 0\\ \frac{x_{k}-x_{b5}}{\sqrt{{\left(x_{k}-x_{b5}\right)}^{2}+{\left(y_{k}-y_{b5}\right)}^{2}}} & \frac{y_{k}-y_{b5}}{\sqrt{{\left(x_{k}-x_{b5}\right)}^{2}+{\left(y_{k}-y_{b5}\right)}^{2}}} & 0\\ \frac{x_{k}-x_{b6}}{\sqrt{{\left(x_{k}-x_{b6}\right)}^{2}+{\left(y_{k}-y_{b6}\right)}^{2}}} & \frac{y_{k}-y_{b6}}{\sqrt{{\left(x_{k}-x_{b6}\right)}^{2}+{\left(y_{k}-y_{b6}\right)}^{2}}} & 0 \end{array}\right].$$

The EKF uses the first-order Taylor expansion to linearly approximate the nonlinear state transition and measurement models. This is where the Jacobian matrices are utilised to represent the first-order partial derivatives of these models for linear approximation [9]. The Jacobian matrices and their inverse (used within the state transition and the measurement models) were generated symbolically using the MATLAB Symbolic toolbox (R2018a, Mathworks, Natick, MA, USA) prior to implementation. The three Jacobian matrices were then solved numerically upon each iteration of the filter. The filter was run offline using the MATLAB Control Systems toolbox.

## 3. Methodology

The methodology implemented for this work consisted of the configuration and calibration of the RTS to set a base reference frame, which was consistently used for all sensor systems throughout the testing procedures. To understand the range error behavior of the UWB system and the linear displacement error of the quadrature encoders, characterisation procedures were executed using the RTS as the ground truth. The results were then implemented into the design of the EKF for the sensor measurement noise and the system input noise variance. Additionally, localisation tests are then carried out to quantify the variation between localisation accuracy of the RTS, custom UWB trilateration algorithm specific to the device and the EKF localisation using range measurements.

### 3.1. Robotic Testing Platform

**Figure 1:**
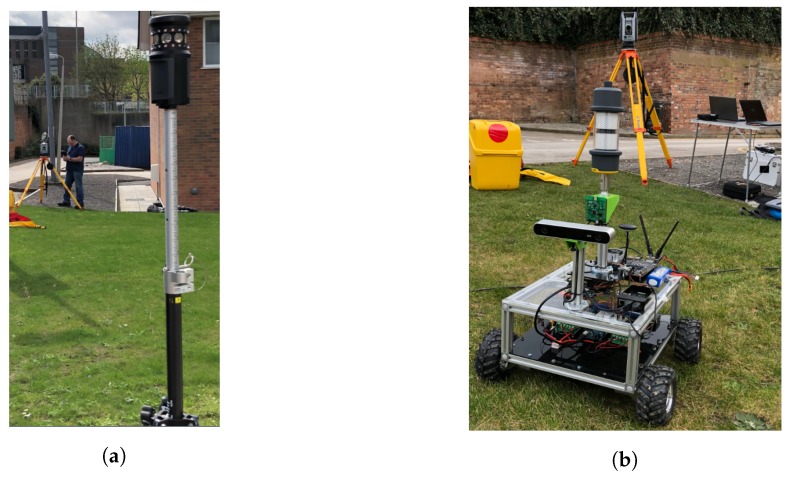
Experimental setup (**a**) backsight prism; (**b**) unmanned developmental platform.

The testbed employed was a skid steer, ridged chassis, four wheeled remotely operated platform similar to the design used in [34]. The platform’s chassis was constructed from 10 mm square extruded aluminium and is driven using four EMG30 DC motors controlled by Quimat DC H-Bridge motor controllers [35]. The full system is powered by a four-cell lithium polymer battery. The motors include built-in incremental quadrature encoders mounted to the motors’ extended shaft. The encoders provide 360 pulses per revolution (ppr), or a pulse every 1° change in angular displacement [35]. The encoders were used to accurately monitor the wheels’ rotation. The on-board processing for data collection, platform control and sensor interaction was achieved using a Nvidia Jetson TX1 (Santa Clara, CA, USA). A Trimble AT360 reflective target was mounted rigidly to the top surface of the platform via a 10 mm extruded aluminium mast allowing for clear line of sight. An image of the robotic platform can be seen in Figure 1.

### 3.2. Robotic Total Station Configuration

**Figure 2:**
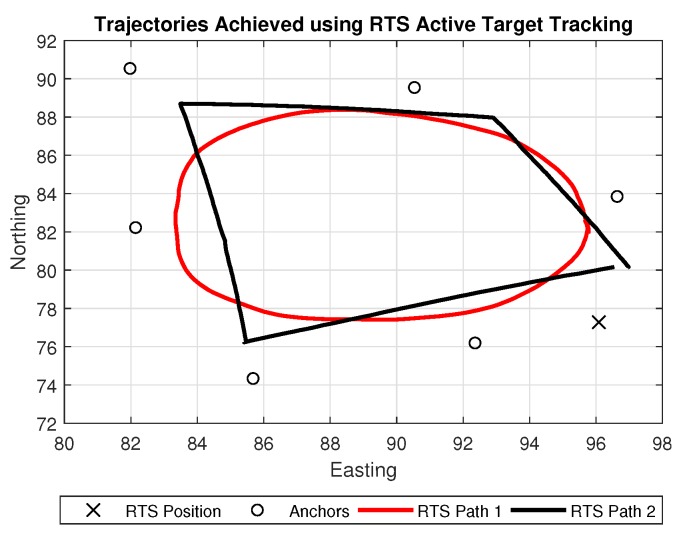
Example trajectories achieved using RTS active target tracking.

The RTS was set up in a static location with calibrated known rectangular Northing, Easting and Elevation coordinates ${X_{RTS}=\left[1996.085,4977.284,198.959\right]}^{T}$. Regarding the British National Grid (BNG) frame, this system allows direct Cartesian output while consistently retaining north reference. The RTS was orientated by taking a back-sight observation measurement to another known point through a centrally mounted telescope to a 360° prism (the back-sight prism and RTS position are shown in Figure 1. Once configured, the TSC3 controller was responsible for operating the RTS through a radio link communication, where the RTS measured both angle and distance to a remote target and the TSC3 calculates the corresponding coordinates of the point within the established reference coordinate system. The RTS was then set to acquire an initial lock prior to robot movement. This enabled a consistent active tracking operation from the RTS using the AT360 target mounted on the robot. Example trajectories produced from the RTS are shown in Figure 2. In order for the trajectory analysis of the RTS to be used as a true baseline in comparison to the other sources of position estimations in this study, it was vital that the real-time NMEA string acquired from the RTS was time synchronised with the rest of the system. This resulted in the generation of a custom Trimble RS232 port monitoring program, which was compatible and able to communicate with other software packages specific to the various implemented localisation systems. This is further covered in Section 3.4 System Level Architecture.

### 3.3. UWB System

The UWB system used for this work is known as Pozyx [36]. Pozyx is a small and lightweight time of flight UWB positioning system that operates using a tag and anchor node network. Each compatible Pozyx transceiver device possesses its own identification code, therefore allowing the mobile operating tag to directly relate a range measurement to a specific anchor. Pozyx firmware deploys a lateration algorithm based on a linear least squares (LLS) method to calculate an estimated location of the tag in relation to the anchor positions. The system configuration settings are split into four sections that cover operating channel, bitrate, pulse repetition frequency (PRF) and preamble length [36]. For this study, the following configuration parameters were used:

- Channel—5,
- Bitrate—110 kbit/s,
- PRF—64 MHz,
- Preamble Length—1024.

The Pozyx firmware positioning algorithm “UWB Positioning Only” was utilised in a 2D mode for metric comparison against the RTS and the results from the EKF.

### 3.4. System Level Architecture

**Figure 3:**
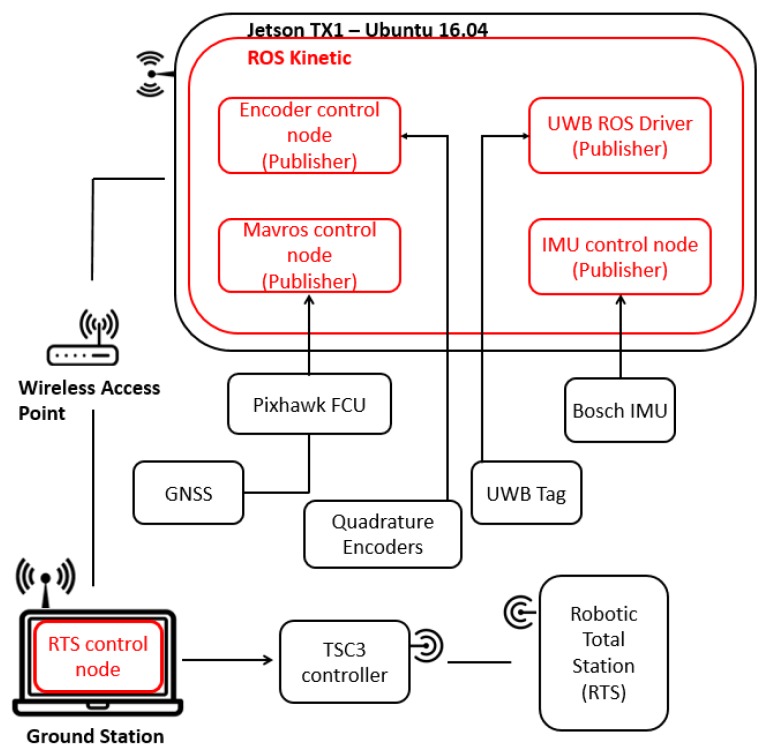
System level architecture.

The system architecture set to operate on-board the robot was designed around the open source Robot Operating System (ROS) framework [37]. ROS is a meta-operating system that enables the integration of multiple sources of information from different devices for the purposes of control. For this work, comparing the trajectory acquired from an RTS with various other sources required precise data synchronisation. Utilising the node based message publishing and subscription protocol that ROS functions through, a custom compatible Trimble software was designed to integrate the RTS with ROS, thereby time synchronising the RTS with other data sources within the system. Another beneficial factor considered when using ROS for the operating framework was the ability to configure a master/slave network that enabled the consistent communication and synchronised data transfer between two different platforms, in this case, a Jetson TX1 (Ubuntu 16.04) (master) and a ground station (Ubuntu 16.04) (slave), where the latter was configured to monitor the incoming pseudo-NMEA GPGAA string acquired from the RTS. Figure 3 shows a graphical overview of the implemented system level architecture.

Dynamic tracking using the RTS is achieved via a lock onto a target, either in the traditional sense using systems such as the Trimble MT1000 (Figure 1a) or aided through high frequency Infrared LED in the case of the Trimble AT360 target (Figure 1b). Although the RTS is capable of tracking MT1000 targets unaided, the prism is equipped with IRLEDs to allow multiple addressed targets to be used in the same process. Figure 1 also shows the RTS with a lock onto the target when mounted on the deployed system. RTS lock is made when a confirmed, stable distance measurement is made to the AT360 target located on the mast of the robot. Once this lock is confirmed, the robot is instructed to follow a predefined set of paths. Data from the RTS is actively collected and stored using the ROSBag functionality of the ROS system along with data collected from the Pozyx and quadrature encoders for post trajectory comparisons.

### 3.5. Range Error Characterisation

In order to characterise the range error of the UWB system, the UWB anchors were placed within a $224\;m^{2}$ environment and RTS was used to take a static measurement directly to the UWB antenna on each anchor to acquire six precise positions all within the BNG coordinate frame. The robot, equipped with the UWB tag and RTS reflective target was then set to execute three paths at a constant speed. This enabled linear interpolation between the UWB and RTS for error analysis. At each update, the robot would receive six distance readings from the UWB anchors and a corresponding position update from the RTS. However, as the true position of the anchors and the RTS are known priors, the acquisition of the true position of the robot using the RTS enabled the calculation of the true Euclidean distance $d_{TA_{i}k}$ between the robot and anchor i:

(15) $$d_{TA_{i}k}=\sqrt[{}]{{\left(x_{TR}-x_{TA_{i}}\right)}^{2}+{\left(y_{TR}-y_{TA_{i}}\right)}^{2}},$$

where $x_{TR_{k}}$, $y_{TR_{k}}$ and $x_{TA_{i}}$, $y_{TA_{i}}$ are the true Cartesian positions of the robot at time *k* and anchor *i*, respectively.

### 3.6. Encoder Error Characterisation

As the encoders were to be used as a control input into the EKF, the noise variance of the input was a required source in the construction of the EKF. Instead of consistently tuning this variable until the EKF yielded expected results, the RTS was used to estimate the noise variance of the encoders. In order to achieve this, the robot was instructed to complete a 10 m track for 10 repetitions. Each 10 m track was split into 2 m segments based on the displacement across ground given from the RTS. This was done to account for any possible wheel slippage occurrence. Time parameters were then cross referenced to identify the displacement estimation given from the encoders at the time corresponding to when the RTS displacement completed each segment. Time alignment between the two sources was achieved through linear interpolation as this test was conducted at a constant speed. The encoder distance and final distance error was then calculated through Equations (16) and (17), respectively:

(16) $$d_{enc}=N_{enc_{k}}Cr,$$

where $N_{enc}$ is the number of encoder pulses at time *k*, *C* is the constant associated with angular displacement per pulse of the encoder set in radians at 0.01745 and *r* is the radius of the wheel:

(17) $$d_{err}=d_{enc}-d_{RTS}.$$

The 10 repetitions of the 10 m track resulted in the error deviation of 0.1185 m. All encoders were assumed to have the same error deviation.

### 3.7. Localisation

The configuration setup concerning the position of the RTS and UWB anchors for the range error characterisation was maintained for the localisation experiments. The results gathered from the EKF localisation algorithm were compared to the trajectory acquired by the RTS and the UWB. The robot was instructed to navigate various types of trajectories, where one was carried out at a constant velocity to allow for linear interpolation for metric positional comparison in terms of (*x*,*y*) between the RTS and the resulting estimations from the EKF. The linearity of the data was assessed to determine its suitability for interpolation. Although the general EKF algorithm performs ideally in pose estimations within the local body frame of the robot, the relationship to a global frame of reference is absent without providing an initial state vector. To convert the operating frame of the EKF to that of the RTS, the initial state vector $X_{0}$ was set to equal the first global position pose acquired from the RTS as shown below:

(18) $$X_{0}=\left[\begin{array}{c} RTS_{x_{0}}\\ RTS_{y_{0}}\\ RTS_{\phi_{0}} \end{array}\right].$$

## 4. Results and Analysis

### 4.1. Range Error Characterisation Results

**Figure 4:**
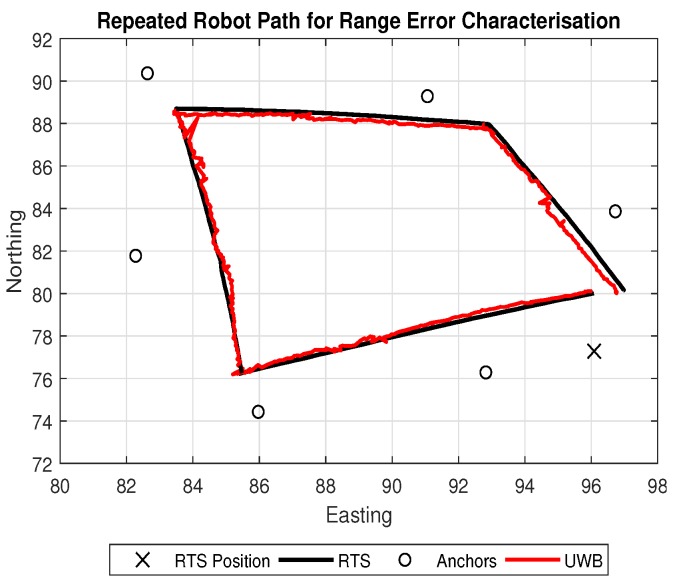
Robot path for Range Error Characterisation.

Characterising the behavior of the range error for the UWB system was carried out with a main focus to determine the precision of the sensor. This is due to the variation of the error being a key input for the EKF. As the robot navigates along the repeated path shown in Figure 4, the distances between the robot and each anchor alternate, providing the opportunity to analyse how the error behaves during a dynamic scenario.

**Figure 5:**
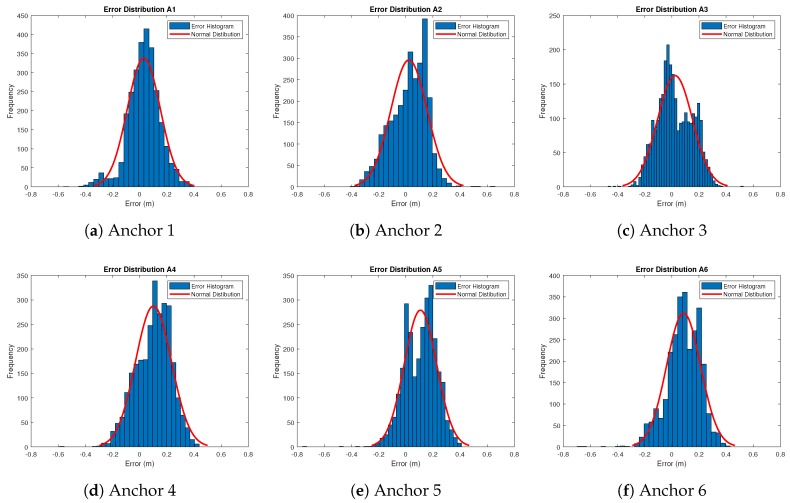
Error Distributions of UWB range measurements.

As each anchor provided a range measurement to the robot, the RTS calculated a corresponding true distance. The errors for each anchor follow a normal distribution shown by the Gaussian plots in Figure 5, where the standard deviation is seen to be similar for each anchor. However, due to the heading of the robot changing, there were notable periods of time where the tag was facing in a contrasting direction to each anchor creating a non clear line of sight condition (NCLOS). These periods may produce an extra source of error in the robot’s path and may possibly increase as larger operating ranges. As the UWB anchor range error is highly dependent upon the environment [38], re-characterisation would be required for each new anchor setup if specific uncertainty estimates for each anchor are used. If a system is to be deployed in a similar configuration and environment, a generalised uncertainty found by taking an average of the specific uncertainties of each anchor may be applicable. However, if the anchor configuration and environment have changed, re-characterisation of the anchors is required. An averaged uncertainty in this form allowed for a generalised uncertainty implementation.

**Figure 6:**
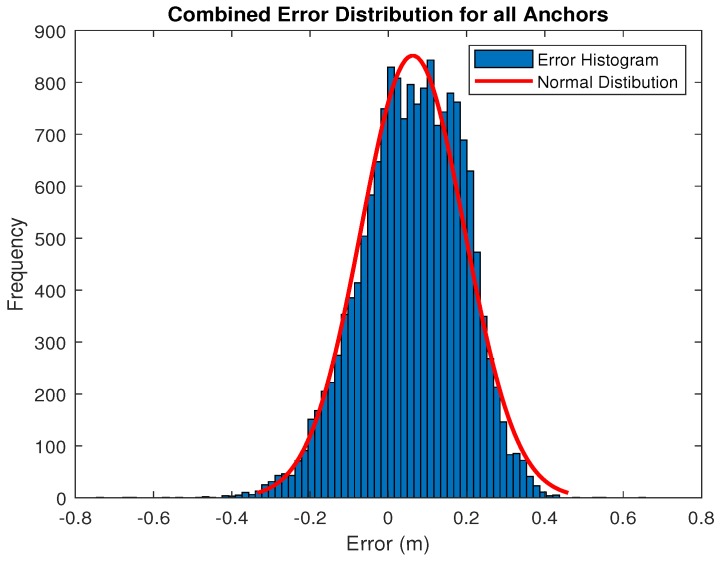
Error distribution for all anchors.

Error results gathered from each anchor during the path were then combined to provide a generalised error metric. The combined errors are represented in Figure 6. All error statistics for each single anchor and the dataset containing combined anchor errors are shown in Table 1.

### 4.2. Localisation Techniques Assessment

**Figure 7:**
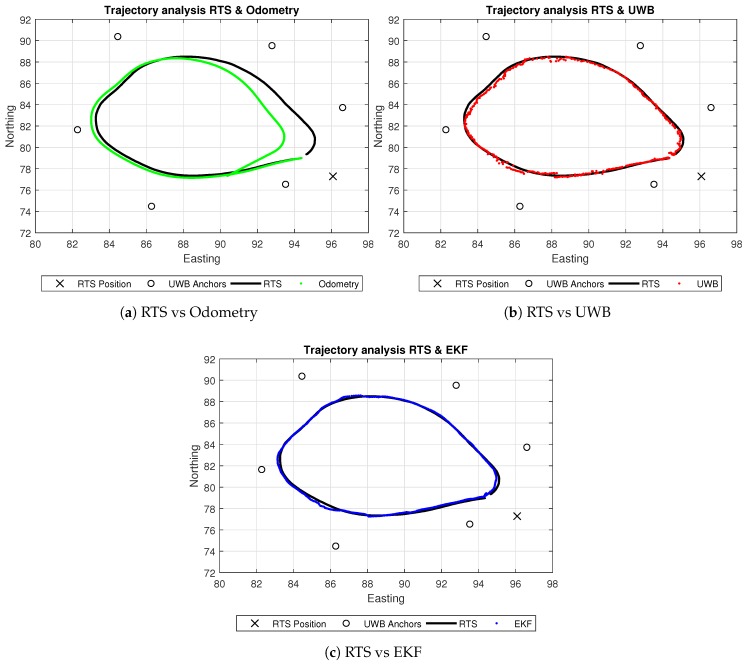
Resulting paths from all techniques and RTS active tracking.

Experiments focusing on the localization outcomes for this work can be seen in Figure 7. Both the EKF and the native UWB system firmware localisation technique produce significantly better results compared to that of the odometry technique, where wheel slippages are clearly noticeable as the robot appears to drift in relation to the true path of the RTS. The path generated from the UWB system is fairly accurate in most areas compared to the ground truth of the RTS. However, in some areas within the environment enclosed by the anchors, the UWB localisation is seen to be slightly noisy, which is something that the path generated by the EKF clearly reduces. Overall, the EKF seems to follow the ground truth trajectory of the RTS in more areas compared to the UWB system. However, it is noticeable that EKF does drift in areas where the UWB seems to be affected by noise. These noisy measurements from the UWB may be a factor in the slight drifts in the EKF, showing that the noise variance of the UWB may in fact be a dynamic variable depending upon the area that the robot is situated within in relation to the external anchor position and coverage.

**Figure 8:**
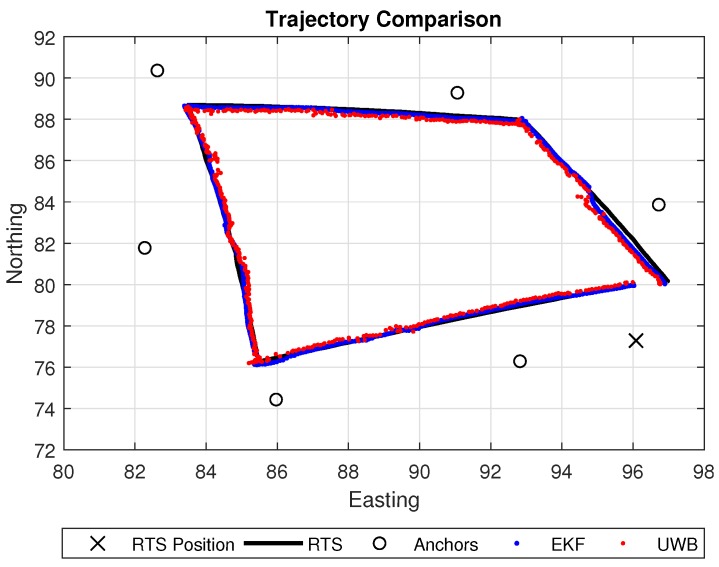
Trajectory comparison between EKF and UWB.

Figure 8 shows the results of the EKF acquired from the robot’s path, which was conducted at a constant speed. Each pose produced from the EKF and UWB as well as corresponding timestamps were used to apply a linear interpolation process on the Northing and Easting plane of the RTS path. This was carried out achieve a point-to-point metric comparison. The EKF again is seen to be less noisy compared to the native UWB localisation algorithm. However, it is also noticeable that the pose of the EKF is seen to drift slightly when the UWB pose estimation from the system’s native algorithm also produces a high level of noise. This is observed directly north in relation to the position of the RTS. This shift in position from the UWB demonstrates again how the variance in noise may be dynamically changing depending upon the environmental conditions at that specific time. Final statistical comparison between the UWB and the EKF on a 2D plane can be found in Table 2. Results show that the EKF achieves a smaller mean error on both the *x*- and *y*-plane and with a reduced error deviation on the *y*, although it yields a slightly increased deviation on the *x*-axis.

## 5. Conclusions

In conclusion, this paper demonstrated the effectiveness of utilising a surveying grade optical instrument, in the form of a robotic total station as a ground truth metric and to dynamically characterise a generalised range uncertainty of a UWB sensor network, all within an intended operating environment. The resulting uncertainty was used as an input into an EKF fused with robot odometry to yield a reduced localisation error metric compared to the UWB system’s native localisation algorithm. Although the odometry is limited when used standalone, when fused with the range measurements from the UWB, it produces a less noisy overall trajectory. However, the EKF is seen to drift in areas within the environment based on the noise increase of the UWB. This has therefore led to planning future work aimed at the inclusion of a generalised adaptive uncertainty metric into the EKF which can also be achieved using the active target tracking feature from the RTS. This is an experiment that cannot be carried out through static tests between two UWB devices as it is environment dependent. The generalised adaptive uncertainty would be taken as a function of the range from the robot to anchor locations, the robot’s position within the environment, and combined with an estimate for the level of obstruction between the robot and anchors dependent upon the robot’s global heading. Additionally, further experimentation will also be undertaken into a specific uncertainty characterisation that may lead to a more effective implementation. This uncertainty will be specific to each individual anchor as opposed to a generalised metric. With this investigation, a comparison study can be completed that assesses the three uncertainty methods: generalised, adaptive generalised and specific, in order to observe which method yields the optimal results.

## Figures and Tables

**Table 1:** Range error statistics for each anchor.

Anchor	Mean Error (m)	Standard Deviation of Error (m)
Anchor 1	0.0301	0.1216
Anchor 2	0.0235	0.1336
Anchor 3	0.0237	0.1287
Anchor 4	0.1014	0.1325
Anchor 5	0.1081	0.1194
Anchor 6	0.0867	0.1256
Combined	0.0622	0.1323

**Table 2:** Positional errors in terms of mean and standard deviation (*x*—Easting, *y*—Northing).

Axis	Mean Error (m)	Standard Deviation of Error (m)
UWB (*x*)	0.0621	0.1478
UWB (*y*)	0.0718	0.1510
EKF (*x*)	0.0167	0.1611
EKF (*y*)	0.0071	0.1326

## Abbreviations

The following abbreviations are used in this manuscript:

EKF	Extended Kalman Filter
TS	Total Station
RTS	Robotic Total Station
GNSS	Global Navigation Satellite System
IMU	Inertial Measurement Unit
INS	Inertial Navigation System
LiDAR	Light Detection and Ranging
UWB	Ultra-Wideband
EDM	Electronic Distance Measurement
RFID	RADIO Frequency Identification
TOA	Time of Arrival
TOF	Time of Flight
BNG	British National Grid
ROS	Robot Operating System
